# Pathology and Treatment of Childbed

**Published:** 1877-08

**Authors:** 


*The Pathology and Treatment of Childbed: A Treatise for Physicians and Students.* By Dr. F. Winckel. Translated by James B. Chadwick, M. D., from the Second German Edition, with additional notes by the author. Philadelphia: Henry C. Lea, 1876.

The work of Dr. Winckel has for some time been favorably known by physicians familiar with the German language, and Dr. Chadwick has now placed the English reading student under many obligations by his excellent translations.

The first thirty-two pages of text are occupied by the Introduction, a summary of the objective appearances in healthy lying-in women, a description of the normal state of the various organs and functions involved in the parturient condition. Following these topics are a few pages devoted to the general etiology and general treatment of chilbed; after which comes the body of the work, commencing with section one, on the puerperal affections of the external and internal organs.

What the author has to say upon the general etiology of puerperal affections may be of interest to our readers as epitomizing to a certain extent his views upon the whole subject. He says at the outset, that everything in woman is in a high degree, predisposed to puerperal affections; quoting and giving additional emphasis to that old statement of Chambond de Montaux, that woman is a being whom nature has, through all ages, allowed to walk upon the edge of an abyss that is ever ready to swallow her up. The causes, he says, of the numerous affections which attend childbred, are principally dependent upon *the changes in the genital organs induced by birth.* Continuing he says: "It is not the location and manner of closing of the placental site, which alone render possible hemorrhages of which we have but slight external signs ; it is the frequent lesions of the cervix uteri, of the vagina, and of the external organs which are peculiarly liable to lead to diseases."

The state of the skin, and intestines come next in the cansideration of the etiology of puerperal diseases, the author considering "catching cold" to be by no means an unimportant factor in the production of disease. Errors in diet and premature physical efforts are next considered, and as something equally necessary with physical rest the author includes complete mental quiet.

Of treatment, Dr. Winkel first mentions the antiphlogistic method. General abstraction of blood he condemns, as not only unnecessary, but often absolutely injurious. In regard to local bleeding, the author says that it may relieve pain, diminish the stasis in the greatly distended capillaries, and relieve passive congestions by freeing the circulation. In practicing local depletion of the circulation, Dr. Winkel does not favor abstracting blood directly from the uterns, but from the contrary, seems to have considerable hesitation in so doing. He uses instead, bleeding from the abdomen, conjoined with the use of cold water, or ice compresses. The author does not exhibit that hesitancy shown by some authors in regard to uterine injections, but recommends their employment in various affections. He recommends Braun's syringe, and gives certain precautions to be observed in making the injections.

Omitting all notice of the various puerperal lesions of the external genital organs and uterus, such as ruptured perineum, inversion of the uterus, etc., which are admirably considered by the author, we proceed at once to examine his treatment of the interesting topic, puerperal fever.

His views on this subject may be briefly stated, by saying that instead of recognizing a puerperal fever, *sui generis*, he considers the febrile accidents of child bed to be all, or nearly all due to septicæmia. Seeing nothing in the diseases to which the term puerperal fever have been applied to distinguish them from surgical septicæmia and pyæmia, except so far as they may be modified by the peculiar condition of the parturient.

We have neither time nor space to follow the author as we would desire, his methods of treatment, while they will be modified in a large degree by American physicians, are in the main excellent. The history of his cases are clearly given, and the deductions logically drawn. We notice one peculiar fact, the ordinary method of measuring by ounces and drachms is frequently used together with the metric system, both being occasionally employed in the same case.

Winckel should be read by all practitioners and students who wish to keep fully informed.

